# Prevalence of Osteoporosis among Adults in a Tertiary Care Hospital: A Descriptive Cross-sectional Study

**DOI:** 10.31729/jnma.4753

**Published:** 2019-12-31

**Authors:** Shriraj Shrestha, Saurav Dahal, Parash Bhandari, Suraj Bajracharya, Anurag Marasini

**Affiliations:** 1 Department of Orthopaedics and Trauma, KIST Medical College and Teaching Hospital, Lalitpur, Nepal

**Keywords:** *body mass index*, *osteoporosis*, *prevalence*

## Abstract

**Introduction:**

Osteoporosis is a common metabolic bone disease characterized by increased bone fragility, yet underdiagnosed and undertreated. With the increase in longevity of the populace, it is becoming an urgent and serious global epidemic. This being a preventable disease, has no clinical manifestations until there is a fracture. Early diagnosis and treatment are of dire necessity. Hence the aim of our study is to find the prevalence of osteoporosis among adults attending a tertiary care hospital.

**Methods:**

This descriptive cross-sectional study was done in a tertiary care hospital, from 15^th^ July 2019 to 15^th^ October 2019 after receiving ethical approval from the Institutional Review Committee (Registration number: 2075/76/119). Convenient sampling was done. Data collection and entry was done in Microsoft excel, point estimate at 95% Confidence Interval was calculated along with frequency and proportion for binary data.

**Results:**

Out of 464 participants, the overall prevalence of osteoporosis was 38 (8.2%) at a 95% Confidence Interval (5.7-10.7%). Among the participants, 141 (30.4%) were male and 323 (69.6%) were female. The mean age of the participant was 41.02±14.96 years.

**Conclusions:**

Prevalence of osteoporosis in our study is high and consistent with other South Asian studies. Early detection of osteoporosis using calcaneal quantitative ultrasound can be a good screening tool.

## INTRODUCTION

Osteoporosis is a disease characterized by low bone mass, deterioration of bone tissue and disruption of bone microarchitecture; leading to compromised bone strength and an increase in the risk of fracture.^1^ It can remain undiagnosed for a long time as it produces no distinct symptoms and is detected when a fracture has already occurred, thus known as a “silent epidemic”.^2^

With an increase in life expectancy, osteoporosis has become an ominous public health problem. Although bone loss is a universal phenomenon, osteoporosis makes bones weak and fragile, there is an increase in the tendency of getting fractured even with trivial trauma.

Such fractures may lead to prolonged morbidity and mortality.^1^ The management of osteoporotic fracture and its complication may consume a significant amount of financial and human resources which can be a huge burden to the nation's health care system.

The aim of the study is to find out the prevalence of osteoporosis among adults attending a tertiary care hospital.

## METHODS

This was a descriptive cross-sectional study conducted in a fortnightly camp arranged in Orthopedic Outpatient department of KIST Medical College and Teaching Hospital in Kathmandu Valley between 15^th^ July 2019 to 15^th^ October 2019 after receiving ethical approval from the Institutional Review Committee (Registration number: 2075/76/119). The participants aged 20 years and above willing to give consent for the study with or without orthopedic problems were included. Participants with old or recent calcaneum fractures or any pathology in the form of tumor or osteomyelitis of right calcaneum were excluded from the study. Convenient sampling was done and the sample size was calculated using the formula,

$$\begin{array}{ll} \text{n} & {\text{=Z}}^{\text{2}}\times \;(\text{p}\times \text{q)}/{\text{e}}^{\text{2}}\\ & \text{=1}{\text{.96}}^{\text{2}}\times \text{0.5}\times \text{(1}-\text{0.5)}/\text{0}{\text{.05}}^{\text{2}}\\ & \text{=0.9604}/\text{0.0025}\\ & \text{=384.16}\\ & \text{=384} \end{array}$$

where,

n= required sample size
p= prevalence of osteoporosis (50%)
q= 1-p
e= margin of error, 5%
Z= 1.96 at 95 % CI

The calculated minimum sample size was 384, however, the total sample size taken was 464. Selection and information bias was minimized as much as possible by collecting data in the appropriately predesigned proforma, point estimated at 95% CI of (5.7-10.7) calculated along with the frequency for binary data.

After explaining the purpose, importance, and procedure in detail a written informed consent was obtained from all the participants. Weight and height were measured with participants in light clothes without shoes by a trained nurse. Weight was measured on an electronic digital scale (rossmax swiss GmbH, Model mo: WB101) and standing height was measured using a portable standiometer. Body mass index (BMI) was calculated as weight (kg) divided by height in meter square (m^2^). Participants were then categorized as underweight (15-19.9), normal weight (20-24.9), overweight (25-29.9) and obese (30-40).^3^

Each participant's demographic data was collected. The participants were made to sit in a chair comfortably. Jelly applied on the medial and lateral aspect of the right heel which was then placed in the machine (Furuno CM-200) with the probes attached so that the scan was performed at the level of the mid-calcaneum. The quality assurance test for the device was performed on each day of screening by a single trained technician. This technique uses the ultrasound waves and measures the broadband ultrasound attenuation (BUA) (dB/MHz) and the speed of sound (SOS) (m/sec) in the center of the bone. The device then combines the values of BUA and SOS to yield a parameter known as the “quantitative ultrasound” (QUS) index, which is expressed as T score.^4^ A T score was calculated as [T score= (subject's BMD - young adult mean BMD / 1 SD of adult mean BMD)] at calcaneum.^5^

According to the T score of BMD assessment by calcaneal QUS, the participants were divided into three groups, namely normal (T score ≥-1.0), Osteopenia (T score between -1.0 and -2.5) and Osteoporosis (T score ≤ -2.5).^6^ The data collected was then analyzed using Statistical Package for the Social Sciences (SPSS) version 23.

## RESULTS

Out of 464 participants, the overall prevalence of osteoporosis was 38 (8.2%) at a 95% Confidence Interval (5.7-10.7). Among the participants 141 (30.4%) were male and 323 (69.6%) were female.

The result was tabulated and analyzed statistically. Among the 38 participants, 9 (23.69%) were males and 29 (76.31%) were females (Table 1).

**Table 1:** Gender distribution in osteoporosis.

Gender	n (%)
Male	9 (23.69)
Female	29 (76.31)
Total	38 (100)

Out of total participants, 137 were in 20-29 years,109 in 30-39 years, 86 in 40-49 years, 57 in 50-59 years, 75 in more than or equal to 60 years group. Out of 38 osteoporotic participants, 0 (0), 2 (5.26%), 1 (2.63%), 12 (31.57%) and 23 (60.52%) were found to be osteoporotic in age group of 20-29 years, 30-39 years, 40-49 years, 50-59 years and in 60 years and more respectively (Table 2).

**Table 2:** Age distribution in osteoporosis.

Age	n (%)
20-29	0 (0)
30-39	2 (5.26)
40-49	1 (2.63)
50-59	12 (31.57)
≥60	23 (60.52)
Total	38 (100)

Out of total participants, 53 (11.4%), 163 (35.1%), 198 (42.7%) and 50 (10.8%) participants were in underweight, normal weight, overweight and obese group respectively. Among the 38 osteoporotic participants, 7 (18.42%), 11 (28.94%), 13 (34.21%), and 7 (18.42%) were osteoporotic in underweight, normal weight, overweight and obese groups respectively (Table 3).

**Table 3:** BMI distribution in osteoporosis.

BMI	n (%)
Under Weight	7 (18.42)
Normal Weight	11 (28.94)
Over Weight	13 (34.21)
Obese	7 (18.42)
Total	38 (100)

## DISCUSSION

The prevalence of osteoporosis in our study was found to be 8.2%, which was similar to a study conducted by Vaishya R, et al. in New Delhi which showed 8.99% prevalence.^7^ Nine percent prevalence of osteoporosis has been reported in northern India.^8^ A study conducted by Haris S, et al. revealed a 30.7% prevalence of osteoporosis among the adult Pakistani population residing in Karachi.^4^ Another study done by Wright N.C, et al. found 10.3% of adults above 50 years residing in the United States to be osteoporotic.^9^ Osteoporosis poses a major health problem associated with significant morbidity and socioeconomic burden.^10^

In our study, the youngest respondent was 20 years and the oldest was 80 years with a mean of 41.02±14.96 years. The maximum number of participants 29.5% were in the age group of 20 to 29 years and 16.2% in the age group for more than 60 years out of total participants. In our study, among the 38 osteoporotic participants, 12 (31.57%) and 23 (60.52%) were found to be osteoporotic in age group of (50-59) years and ≥60 years respectively. This showed an increase in osteoporosis with increasing age after the age of 50 years which was also supported by a similar study conducted by Wright, et al. revealing 3.4% at the age of 50-59 years and 10.9% at the age of 80 or more. A Srilankan study on the prevalence of osteoporosis in the urban population aged more than 50 years showed osteoporosis in 27% of women and 7% of men and in subjects less than 50 years osteoporosis was seen in just 9% of women and 3% of men.^11^

Osteoporosis, although is mislabeled as a women's disease by the public, can affect both men and women equally.^12^ Among the 38 osteoporotic participants, 29 (76.34%) were found to be female and 9 (23.68%) were male. This result was quite alarming to the male population who can also be affected reasonably. Studies have shown that bone loss starts from 35-40 years both in men and women. In men, a small longitudinal bone loss is observed throughout life, whereas in women there is an additional bone loss in association with estrogen insufficiency during peri-menopausal and post-menopausal period.^13^ A study carried out by Hernlund E, et al. found out 21% of females and 6% of males residing in Sweden to be osteoporotic. This study showed 3-4 times more osteoporosis in the female population than in the male.^14^ In contrast to the above study, Chitten JJ, et al. from India showed a prevalence of 8.6% and 5.8% osteoporosis in men and women respectively, in the age group of 40-59years.^15^

The prevalence of osteoporosis is increasing day by day and so is the obesity, a study conducted in the United States to estimate whether the higher prevalence of overweight (increase in BMI) was likely to reduce osteoporosis among older women, they concluded that overweight (high BMI) was not a protective factor for osteoporosis.^16^ In our study the mean height of the participants was 154.93±8.75cm and weight was 60.77±11.52kg.

In our study, among the total participants, 53 (11.4%) were underweight, 163 (35.1%) were normal weight, 198 (42.7%) were overweight and 50 (10.8%) were obese. Among the 38 osteoporotic participants, 7 (18.42%) were in underweight group, 1 (28.94%) were in normal weight group, 13 (34.21%) were in overweight group and 7 (18.42%) were in obese group. Normal and overweight participants were found to be relatively more osteoporotic i.e. 28.94% and 34.21% respectively which was similar to a study conducted by Harris, et al.^4^

Diagnosis of osteoporosis using dual-energy x-ray absorptiometry (DEXA) machine is regarded as the gold standard, but its availability only in a few centers of big cities with high cost and radiation hazard has limited its use. A study conducted by Rajouria A, et al. in Bir Hospital, Nepal compared bone density using quantitative ultrasound (QUS) and DEXA scan, which showed significant co-relation in t-score, thereby concluding QUS as a sensitive screening tool to detect the change in bone mass.^17,18^ Because of the limited availability and affordability of DEXA in our country, we have used heel ultrasound (QUS) to determine BMD as it is easy to use, economical, portable and without radiation hazard.

Even though there are radiographic diagnostic criteria to define osteoporosis, it cannot be used as a screening tool because of its high cost and unnecessary exposure to radiation. Clinical diagnosis of osteoporosis can be done in subjects over the age of 50 years by an increase in thoracic kyphosis or diminished in height. Both these methods need to be confirmed by the assessment of BMD using DEXA or calcaneal QUS.

If osteoporosis is not managed properly in time it may result in fragile or insufficiency fracture which is commonly seen in the vertebra, hip and distal forearm (Colle's). Once the (fragile) fracture is encountered, apart from the financial burden to the family and society there is a requirement of long term nursing care, decrease in quality of life, social isolation, depression, and loss of self-esteem.^19^

Osteoporosis is a treatable condition, where one can decrease the morbidity and mortality among the elderly by adequate intake of calcium, vitamin D (adequate exposure to sunlight), bisphosphonates and newer anabolic agent like Teriparatide (recombinant human PTH).^12^ Thirty minutes of weight bearing exercises like walking and back posture exercises should be advocated throughout life. Measures should be taken to prevent fall injury in those who are diagnosed as osteoporosis.

The limitation of the study is that since the study was done in small sample size and convenient sampling was done, the findings of the study can't be generalized. And also, the cause and risk factors for osteoporosis have not been identified in this study, hence further study of higher level of evidence with random sampling technique and in larger settings is recommended; thereby providing national data on the prevalence of osteoporosis in Nepal.

## CONCLUSIONS

The prevalence of osteoporosis in our study is high and is consistent with other studies of our neighboring countries like India, Pakistan, and Srilanka. Since osteoporosis is a preventable disease if identified and treated early, regular screening of individuals above 50 years with effective management at low cost can decrease the nation's burden and improve the quality of life even in declining age.

## Conflict of Interest

**None.**
